# Current Awareness on Comparative and Functional Genomics

**DOI:** 10.1002/cfg.229

**Published:** 2003-07


**Comparative and Functional Genomics**
*Comp Funct Genom* 2003; **4**: 450-457
Published online in Wiley InterScience (www.interscience.wiley.com). **DOI:** 10.1002/cfg.229

### Current awareness on comparative and functional genomics

### 1 Reviews & symposia

2002. Special issue: Papers from the Symposium on Genomics & Proteomics: Impact on Medicine and Health, Asian Medical Center, Seoul, Korea. *Genet Med* **4:** (6)
2003. Special issue: BioXAS study weekend on contribution of BioXAS to structural genomics: Developments in Theory and Refinement, LURE, Orsay, France, 3 June-1 July 2001. *J Synchrotron Radiat* **10:** (1)
2003. Special issue: Chloroplasts and Mitochondria - Functional Genomics and Evolution. Meetings held at the Royal Society, London, England on 26 and 27 June 2002 and at The Novartis Foundation on 28 June 2002. *Philos Trans R Soc Lond B* **358:** (1429)
2003. Special issue: The Molecular Biology Database Collection - 2003 update. *Nucleic Acids Res* **31:** (1)
Ali A, Crawford MJ. 2002. Developmental biology: An array of new possibilities. *Biotechnol Advan* **20:** (5-6) 363.
Anantharaman V, Aravind L, Koonin EV. 2003. Emergence of diverse biochemical activities in evolutionarily conserved structural scaffolds of proteins. *Curr Opin Chem Biol* **7:** (1) 12.
Bauer A, Kuster B. 2003. Affinity purification-mass spectrometry: Powerful tools for the characterization of protein complexes (Review). *Eur J Biochem* **270:** (4) 570.
Boguski MS, McIntosh MW. 2003. Biomedical informatics for proteomics. *Nature* **422:** (6928) 233.
Buchanan SG. 2002. Structural genomics: Bridging functional genomics and structure-based design. *Curr Opin Drug Discov Dev* **5:** (3) 367.
Bugelski PJ. 2002. Gene expression profiling for pharmaceutical toxicology screening. *Curr Opin Drug Discov Dev* **5:** (1) 79.
Celis JE, Gromov P. 2003. Proteomics in translational cancer research: Toward an integrated approach. *Cancer Cell* **3:** (1) 9.
Chin KV, Kong ANT. 2002. Application of DNA microarrays in pharmacogenomics and toxicogenomics. *Pharmaceut Res* **19:** (12) 1773.
Conway T, Schoolnik GK. 2003. Microarray expression profiling: Capturing a genome-wide portrait of the transcriptome (Review). *Mol Microbiol* **47:** (4) 879.
Cutler P. 2003. Protein arrays: The current state-of-the-art. *Proteomics* **3:** (1) 3.
Dreger M. 2003. Emerging strategies in mass-spectrometry based proteomics (Review). *Eur J Biochem* **270:** (4) 569.
Dreger M. 2003. Proteome analysis at the level of subcellular structures (Review). *Eur J Biochem* **270:** (4) 589.
Falb D, Jindal S. 2002. Chemical genomics: Bridging the gap between the proteome and therapeutics. *Curr Opin Drug Discov Dev* **5:** (4) 532.
Fernie AR. 2003. Metabolome characterisation in plant system analysis. *Funct Plant Biol* **30:** (1) 111.
Ffrench-Constant R, Waterfield N, Daborn P, Joyce S, Bennett H, Au C, Dowling A, Boundy S, Reynolds S, Clarke D. 2003. *Photorhabdus*: Towards a functional genomic analysis of a symbiont and pathogen. *FEMS Microbiol Rev* **26:** (5) 433.
Fiehn O, Weckwerth W. 2003. Deciphering metabolic networks (Review). *Eur J Biochem* **270:** (4) 579.
Frazer KA, Elnitski L, Church DM, Dubchak I, Hardison RC. 2003. Cross-species sequence comparisons: A review of methods and available resources. *Genome Res* **13:** (1) 1.
Gavin AC, Superti-Furga G. 2003. Protein complexes and proteome organization from yeast to man. *Curr Opin Chem Biol* **7:** (1) 21.
Gerling IC, Solomon SS, Bryer-Ash M. 2003. Genomes, transcriptomes, and proteomes: Molecular medicine and its impact on medical practice. *Arch Intern Med* **163:** (2) 190.
Goshe MB, Smith RD. 2003. Stable isotope-coded proteomic mass spectrometry. *Curr Opin Biotechnol* **14:** (1) 101.
Guo QM. 2003. DNA microarray and cancer. *Curr Opin Oncol* **15:** (1) 36.
Haberkorn U, Altmann A. 2002. Radionuclide imaging in the post-genomic era. *J Cell Biochem* **87:** (Suppl 39) 1.
Hatfield GW, Hung SP, Baldi P. 2003. Differential analysis of DNA microarray gene expression data (Review). *Mol Microbiol* **47:** (4) 871.
Hermeking H. 2003. Serial analysis of gene expression and cancer. *Curr Opin Oncol* **15:** (1) 44.
Herniou EA, Olszewski JA, Cory JS, O'Reilly DR. 2003. The genome sequence and evolution of baculoviruses. *Annu Rev Entomol* (48) 211.
Jeffery DA, Bogyo M. 2003. Chemical proteomics and its application to drug discovery. *Curr Opin Biotechnol* **14:** (1) 87.
Kalume DE, Molina H, Pandey A. 2003. Tackling the phosphoproteome: Tools and strategies. *Curr Opin Chem Biol* **7:** (1) 64.
Kingsmore SF, Patel DD. 2003. Multiplexed protein profiling on antibody-based microarrays by rolling circle amplification. *Curr Opin Biotechnol* **14:** (1) 74.
Kozarich JW. 2003. Activity-based proteomics: Enzyme chemistry redux. *Curr Opin Chem Biol* **7:** (1) 78.
Lan N, Montelione GT, Gerstein M. 2003. Ontologies for proteomics: Towards a systematic definition of structure and function that scales to the genome level. *Curr Opin Chem Biol* **7:** (1) 44.
Lescuyer P, Strub JM, Luche S, Diemer H, Martinez P, Van Dorsselaer A, Lunardi J, Rabilloud T. 2003. Progress in the definition of a reference human mitochondrial proteome. *Proteomics* **3:** (2) 157.
Liu ET. 2003. Classification of cancers by expression profiling. *Curr Opin Genet Dev* **13:** (1) 97.
Liu JF, Rost B. 2003. Domains, motifs and clusters in the protein universe. *Curr Opin Chem Biol* **7:** (1) 5.
Lokey RS. 2003. Forward chemical genetics: Progress and obstacles on the path to a new pharmacopoeia. *Curr Opin Chem Biol* **7:** (1) 91.
Mann M, Jensen ON. 2003. Proteomic analysis of post-translational modifications (Review). *Nat Biotechnol* **21:** (3) 255.
Mao JH, Balmain A. 2003. Genomic approaches to identification of tumour-susceptibility genes using mouse models. *Curr Opin Genet Dev* **13:** (1) 14.
Martin AC, Drubin DG. 2003. Impact of genome-wide functional analyses on cell biology research. *Curr Opin Cell Biol* **15:** (1) 6.
Meyer UA, Gut J. 2002. Genomics and the prediction of xenobiotic toxicity. *Toxicology* **181-182:** 463.
Neumann NF, Galvez F. 2002. DNA microarrays and toxicogenomics: Applications for ecotoxicology? *Biotechnol Advan* **20:** (5-6) 391.
O'Neill GM, Catchpoole DR, Golemis EA. 2003. From correlation to causality: Microarrays, cancer, and cancer treatment. *Biotechniques* (Suppl) 64.
Pennie WD. 2002. Custom cDNA microarrays, technologies and applications. *Toxicology* **181-182:** 551.
Petersen M, Wengel J. 2003. LNA: A versatile tool for therapeutics and genomics (Review). *Trends Biotechnol* **21:** (2) 74.
Pollock JD. 2002. Gene expression profiling: Methodological challenges, results, and prospects for addiction research. *Chem Phys Lipids* **121:** (1-2) 241.
Salter AH, Nilsson KC. 2002. Informatics and multivariate analysis of toxicogenomics data. *Curr Opin Drug Discov Dev* **5:** (6) 117.
Sechi S, Oda Y. 2003. Quantitative proteomics using mass spectrometry. *Curr Opin Chem Biol* **7:** (1) 70.

In order to keep subscribers up-to-date with the latest developments in their field, this current awareness service is provided by John Wiley & Sons and contains newly-published material on comparative and functional genomics. Each bibliography is divided into 16 sections. 1 Reviews & symposia; 2 General; 3 Large-scale sequencing and mapping; 4 Evolutionary genomics; 5 Comparative genomics; 6 Pathways, gene families and regulons; 7 Pharmacogenomics; 8 EST, cDNA and other clone resources; 9 Functional genomics; 10 Transcriptomics; 11 Proteomics; 12 Protein structural genomics; 13 Metabolomics; 14 Genomic approaches to development; 15 Technological advances; 16 Bioinformatics. Within each section, articles are listed in alphabetical order with respect to author. If, in the preceding period, no publications are located relevant to any one of these headings, that section will be omitted.

Copyright © 2003 John Wiley & Sons, Ltd.

Sehgal A. 2002. Drug discovery and development using chemical genomics. *Curr Opin Drug Discov Dev* **5:** (4) 526.
Sellers TA, Yates JR. 2003. Review of proteomics with applications to genetic epidemiology. *Genet Epidemiol* **24:** (2) 83.
Sidhu SS, Bader GD, Boone C. 2003. Functional genomics of intracellular peptide recognition domains with combinatorial biology methods. *Curr Opin Chem Biol* **7:** (1) 97.
Stein TP, Wade CE. 2003. Protein turnover in atrophying muscle: From nutritional intervention to microarray expression analysis. *Curr Opin Clin Nutr Metab Care* **6:** (1) 95.
Storck T, Von Brevern MC, Behrens CK, Scheel J, Bach A. 2002. Transcriptomics in predictive toxicology. *Curr Opin Drug Discov Dev* **5:** (1) 90.
Tao WA, Aebersold R. 2003. Advances in quantitative proteomics *via* stable isotope tagging and mass spectrometry. *Curr Opin Biotechnol* **14:** (1) 110.
Taylor SW, Fahy E, Ghosh SS. 2003. Global organellar proteomics (Review). *Trends Biotechnol* **21:** (2) 82.
Thomas RS, Rank DR, Penn SG, Zastrow GM, Hayes KR, Hu TH, Pande K, Lewis M, Jovanovich SB, Bradfield CA. 2002. Application of genomics to toxicology research. *Environ Health Perspect* **110:** (Suppl 6) 919.
Turk BE, Cantley LC. 2003. Peptide libraries: At the crossroads of proteomics and bioinformatics. *Curr Opin Chem Biol* **7:** (1) 84.
Tyers M, Mann M. 2003. From genomics to proteomics. *Nature* **422:** (6928) 193.
Waldo GS. 2003. Genetic screens and directed evolution for protein solubility. *Curr Opin Chem Biol* **7:** (1) 33.
Wiseman SB, Singer TD. 2002. Applications of DNA and protein microarrays in comparative physiology. *Biotechnol Advan* **20:** (5-6) 379.
Wu CC, Yates JR. 2003. The application of mass spectrometry to membrane proteomics (Review). *Nat Biotechnol* **21:** (3) 262.
Yang CH, Bakshi BR, Rathman JF, Blower PE. 2002. Multiscale and Bayesian approaches to data analysis in genomics high-throughput screening. *Curr Opin Drug Discov Dev* **5:** (3) 428.
Yokoyama S. 2003. Protein expression systems for structural genomics and proteomics. *Curr Opin Chem Biol* **7:** (1) 39.
Yu JW, Lemmon MA. 2003. Genome-wide analysis of signaling domain function. *Curr Opin Chem Biol* **7:** (1) 103.
Zendman AJW, Ruiter DJ, Van Muijen GNP. 2003. Cancer/testis-associated genes: Identification, expression profile, and putative function (Review). *J Cell Physiol* **194:** (3) 272.
Zhang C, Kim SH. 2003. Overview of structural genomics: From structure to function. *Curr Opin Chem Biol* **7:** (1) 28.
Zhu H, Snyder M. 2003. Protein chip technology. *Curr Opin Chem Biol* **7:** (1) 55.

### 3 Large-scale sequencing and mapping

Bentley SD, Maiwald M, Murphy LD, Pallen MJ, Yeats CA, Dover LG, Norbertczak HT, Besra GS, Quail MA, Harris DE *et al*. 2003. Sequencing and analysis of the genome of the Whipple's disease bacterium *Tropheryma whipplei*. *Lancet* **361:** (9358) 637.
Bruggemann H, Baumer S, Fricke WF, Wiezer A, Liesegang H, Decker I, Herzberg C, Martinez-Arias R, Merkl R, Henne A *et al*. 2003. The genome sequence of *Clostridium tetani*, the causative agent of tetanus disease. *Proc Natl Acad Sci U S A* **100:** (3) 1316.
Flicek P, Keibler E, Hu P, Korf I, Brent MR. 2003. Leveraging the mouse genome for gene prediction in human: From whole-genome shotgun reads to a global synteny map. *Genome Res* **13:** (1) 46.
Kaneko T, Nakamura Y, Sato S, Minamisawa K, Uchiumi T, Sasamoto S, Watanabe A, Idesawa K, Iriguchi M, Kawashima K *et al*. 2002. Complete genomic sequence of nitrogen-fixing symbiotic bacterium *Bradyrhizobium japonicum* USDA110. *DNA Res* **9:** (6) 189.
Kessler MM, Zeng QD, Hogan S, Cook R, Morales AJ, Cottarel G. 2003. Systematic discovery of new genes in the *Saccharomyces cerevisiae* genome. *Genome Res* **13:** (2) 264.
Kleerebezem M, Boekhorst J, Van Kranenburg R, Molenaar D, Kuipers OP, Leer R, Tarchini R, Peters SA, Sandbrink HM, Fiers MWEJ *et al*. 2003. Complete genome sequence of *Lactobacillus plantarum* WCFS1. *Proc Natl Acad Sci U S A* **100:** (4) 1990.
Makino K, Oshima K, Kurokawa K, Yokoyama K, Uda T, Tagomori K, Iijima Y, Najima M, Nakano M, Yamashita A *et al*. 2003. Genome sequence of *Vibrio parahaemolyticus*: A pathogenic mechanism distinct from that of *V. cholerae*. *Lancet* **361:** (9359) 743.
Meinke DW, Meinke LK, Showalter TC, Schissel AM, Mueller LA, Tzafrir I. 2003. A sequence-based map of *Arabidopsis* genes with mutant phenotypes. *Plant Physiol* **131:** (2) 409.

### 4 Evolutionary genomics

Bon E, Casaregola S, Blandin G, Llorente B, Neuveglise C, Munsterkotter M, Guldener U, Mewes HW, Van Helden J, Dujon B, Gaillardin C. 2003. Molecular evolution of eukaryotic genomes: Hemiascomycetous yeast spliceosomal introns. *Nucleic Acids Res* **31:** (4) 1121.
Chattopadhyay S, Sahoo S, Kanner WA, Chakrabarti J. 2003. Pressures in archaeal protein coding genes: A comparative study. *Comp Funct Genom* **4:** (1) 56.
Pevzner P, Tesler G. 2003. Genome rearrangements in mammalian evolution: Lessons from human and mouse genomes. *Genome Res* **13:** (1) 37.
Van Ham RCHJ, Kamerbeek J, Palacios C, Rausell C, Abascal F, Bastolla U, Fernandez JM, Jimenez L, Postigo M, Silva FJ *et al*. 2003. Reductive genome evolution in *Buchnera aphidicola*. *Proc Natl Acad Sci U S A* **100:** (2) 581.

### 5 Comparative genomics

Bergman NH, Akerley BJ. 2003. Position-based scanning for comparative genomics and identification of genetic islands in *Haemophilus influenzae* type b. *Infect Immun* **71:** (3) 1098.
Call DR, Borucki MK, Besser TE. 2003. Mixed-genome microarrays reveal multiple serotype and lineage-specific differences among strains of *Listeria monocytogenes*. *J Clin Microbiol* **41:** (2) 632.
Chastanet A, Fertil J, Msadek T. 2003. Comparative genomics reveal novel heat shock regulatory mechanisms in *Staphylococcus aureus* and other Gram-positive bacteria. *Mol Microbiol* **47:** (4) 1061.
Couronne O, Poliakov A, Bray N, Ishkhanov T, Ryaboy D, Rubin E, Pachter L, Dubchak I. 2003. Strategies and tools for whole-genome alignments. *Genome Res* **13:** (1) 73.
Guigo R, Dermitzakis ET, Agarwal P, Ponting CP, Parra G, Reymond A, Abril JF, Keibler E, Lyle R, Ucla C *et al*. 2003. Comparison of mouse and human genomes followed by experimental verification yields an estimated 1,019 additional genes. *Proc Natl Acad Sci U S A* **100:** (3) 1140.
Gutacker MM, Smoot JC, Migliaccio CAL, Ricklefs SM, Hua S, Cousins DV, Graviss EA, Shashkina E, Kreiswirth BN, Musser JM. 2002. Genome-wide analysis of synonymous single nucleotide polymorphisms in *Mycobacterium tuberculosis* complex organisms: Resolution of genetic relationships among closely related microbial strains. *Genetics* **162:** (4) 1533.
Spencer DH, Kas A, Smith EE, Raymond CK, Sims EH, Hastings M, Burns JL, Kaul R, Olson MV. 2003. Whole-genome sequence variation among multiple isolates of *Pseudomonas aeruginosa*. *J Bacteriol* **185:** (4) 1316.
Van Leeuwen H, Monfort A, Zhang HB, Puigdomenech P. 2003. Identification and characterisation of a melon genomic region containing a resistance gene cluster from a constructed BAC library. Microcolinearity between *Cucumis melo* and *Arabidopsis thaliana*. *Plant Mol Biol* **51:** (5) 703.
Van Sluys MA, De Oliveira MC, Monteiro-Vitorello CB, Miyaki CY, Furlan LR, Camargo LEA, Da Silva ACR, Moon DH, Takita MA, Lemos EGM *et al*. 2003. Comparative analyses of the complete genome sequences of Pierce's disease and citrus variegated chlorosis strains of *Xylella fastidiosa*. *J Bacteriol* **185:** (3) 1018.
Woram RA, Gharbi K, Sakamoto T, Hoyheim B, Holm LE, Naish K, McGowan C, Ferguson MM, Phillips RB, Stein J *et al*. 2003. Comparative genome analysis of the primary sex-determining locus in salmonid fishes. *Genome Res* **13:** (2) 272.

### 6 Pathways, gene families and regulons

Antoine R, Jacob-Dubuisson F, Drobecq H, Willery E, Lesjean S, Locht C. 2003. Overrepresentation of a gene family encoding extracyto-

Copyright © 2003 John Wiley & Sons, Ltd. 

plasmic solute receptors in *Bordetella*. *J Bacteriol* **185:** (4) 1470.
Mader U, Antelmann H, Buder T, Dahl MK, Hecker M, Homuth G. 2002. *Bacillus subtilis* functional genomics: Genome-wide analysis of the DegS-DegU regulon by transcriptomics and proteomics. *Mol Genet Genomics* **268:** (4) 455.
Ramirez-Parra E, Frundt C, Gutierrez C. 2003. A genome-wide identification of E2F-regulated genes in *Arabidopsis*. *Plant J* **33:** (4) 801.

### 7 Pharmacogenomics

Adam PJ, Boyd R, Tyson KL, Fletcher GC, Stamps A, Hudson L, Poyser HR, Redpath N, Griffiths M, Steers G *et al*. 2003. Comprehensive proteomic analysis of breast cancer cell membranes reveals unique proteins with potential roles in clinical cancer. *J Biol Chem* **278:** (8) 6482.
Amin RP, Hamadeh HK, Bushel PR, Bennett L, Afshari CA, Paules RS. 2002. Genomic interrogation of mechanism(s) underlying cellular responses to toxicants. *Toxicology* **181-182:** 555.
Anders RA, Yerian LM, Tretiakova M, Davison JM, Quigg RJ, Domer PH, Hoberg J, Hart J. 2003. cDNA microarray analysis of macroregenerative and dysplastic nodules in end-stage hepatitis C virus-induced cirrhosis. *Am J Pathol* **162:** (3) 991.
Andersen H, Jensen ON, Moiseeva EP, Eriksen EF. 2003. A proteome study of secreted prostatic factors affecting osteoblastic activity: Galectin-1 is involved in differentiation of human bone marrow stromal cells. *J Bone Miner Res* **18:** (2) 195.
Aoyama I, Enomoto A, Niwa T. 2003. Effects of oral adsorbent on gene expression profile in uremic rat kidney: cDNA array analysis. *Am J Kidney Dis* **41:** (3 Suppl 1) S8.
Astier AL, Xu RH, Svoboda M, Hinds E, Munoz O, De Beaumont R, Crean CD, Gabig T, Freedman AS. 2003. Temporal gene expression profile of human precursor B leukemia cells induced by adhesion receptor: Identification of pathways regulating B-cell survival. *Blood* **101:** (3) 1118.
Bandow JE, Brotz H, Leichert LIO, Labischinski H, Hecker M. 2003. Proteomic approach to understanding antibiotic action. *Antimicrob Agents Chemother* **47:** (3) 948.
Choi J, Conrad CC, Dai R, Malakowsky CA, Talent JM, Carroll CA, Weintraub ST, Gracy RW. 2003. Vitamin E prevents oxidation of antiapoptotic proteins in neuronal cells. *Proteomics* **3:** (1) 73.
Choquet-Kastylevsky G. 2002. Diagnosis of prostate cancer (French). *Biofutur* (Suppl 1) 39.
Chua MS, Barry C, Chen X, Salvatierra O, Sarwal MM. 2003. Molecular profiling of anemia in acute renal allograft rejection using DNA microarrays. *Am J Transplant* **3:** (1) 17.
Cobb JP, Laramie JM, Stormo GD, Morrissey JJ, Shannon WD, Qiu YY, Karl IE, Buchman TG, Hotchkiss RS. 2002. Sepsis gene expression profiling: Murine splenic compared with hepatic responses determined by using complementary DNA microarrays. *Crit Care Med* **30:** (12) 2711.
Coyle B, Freathy C, Gant TW, Roberts RA, Cain K. 2003. Characterization of the transforming growth factor-$\beta_1$-induced apoptotic transcriptome in FaO hepatoma cells. *J Biol Chem* **278:** (8) 5920.
Davidsson P, Sjogren M, Andreasen N, Lindbjer M, Nilsson CL, Westman-Brinkmalm A, Blennow K. 2002. Studies of the pathophysiological mechanisms in frontotemporal dementia by proteome analysis of CSF proteins. *Mol Brain Res* **109:** (1-2) 128.
Dayem MA, Moreilhon C, Turchi L, Magnone V, Christen R, Ponzio G, Barbry P. 2003. Early gene expression in wounded human keratinocytes revealed by DNA microarray analysis. *Comp Funct Genom* **4:** (1) 47.
De Torres M, Sanchez P, Fernandez-Delmond I, Grant M. 2003. Expression profiling of the host response to bacterial infection: The transition from basal to induced defence responses in RPM1-mediated resistance. *Plant J* **33:** (4) 665.
De Vos S, Krug U, Hofmann WK, Pinkus GS, Swerdlow SH, Wachsman W, Grogan TM, Said JW, Koeffler HP. 2003. Cell cycle alterations in the blastoid variant of mantle cell lymphoma (MCL-BV) as detected by gene expression profiling of mantle cell lymphoma (MCL) and MCL-BV. *Diagn Mol Pathol* **12:** (1) 35.
Fountoulakis M, Schlaeger EJ. 2003. The mitochondrial proteins of the neuroblastoma cell line IMR-32. *Electrophoresis* **24:** (1-2) 260.
Giordano TJ, Thomas DG, Kuick R, Lizyness M, Misek DE, Smith AL, Sanders D, Aljundi RT, Gauger PG, Thompson NW *et al*. 2003. Distinct transcriptional profiles of adrenocortical tumors uncovered by DNA microarray analysis. *Am J Pathol* **162:** (2) 521.
Gonzalez LJ, Castellanos-Serra L, Badock V, Diaz M, Moro A, Perea S, Santos A, Paz-Lago D, Otto A, Muller EC *et al*. 2003. Identification of nuclear proteins of small cell lung cancer cell line H82: An improved procedure for the analysis of silver-stained proteins. *Electrophoresis* **24:** (1-2) 237.
Gu S, Ripp SL, Prough RA, Geoghegan TE. 2003. Dehydroepiandrosterone affects the expression of multiple genes in rat liver including 11β-hydroxysteroid dehydrogenase type 1: A cDNA array analysis. *Mol Pharmacol* **63:** (3) 722.
Hanash S. 2003. Disease proteomics. *Nature* **422:** (6928) 226.
He C. 2003. Proteomic analysis of human bronchoalveolar lavage fluid: expression profiling of surfactant-associated protein A isomers derived from human pulmonary alveolar proteinosis using immunoaffinity detection. *Proteomics* **3:** (1) 87.
Higgins JPT, Shinghal R, Gill H, Reese JH, Terris M, Cohen RJ, Fero M, Pollack JR, Van de Rijn M, Brooks JD. 2003. Gene expression patterns in renal cell carcinoma assessed by complementary DNA microarray. *Am J Pathol* **162:** (3) 925.
Higuchi E, Oridata N, Furuta Y, Suzuki S, Hatakeyama H, Sawa H, Sunayashiki-Kusuzaki K, Yamazaki K, Inuyama Y, Fukuda S. 2003. Differentially expressed genes associated with *cis*-diamminedichloroplatinum (II) resistance in head and neck cancer using differential display and cDNA microarray. *Head Neck J* **25:** (3) 187.
Hirakawa S, Hong YK, Harvey N, Schacht V, Matsuda K, Libermann T, Detmar M. 2003. Identification of vascular lineage-specific genes by transcriptional profiling of isolated blood vascular and lymphatic endothelial cells. *Am J Pathol* **162:** (2) 575.
Hondermarck H. 2002. Proteomics of breast cancer (French). *Biofutur* (Suppl 1) 43.
Huerta S, Harris DM, Jazirehi A, Bonavida B, Elashoff D, Livingston EH, Heber D. 2003. Gene expression profile of metastatic colon cancer cells resistant to cisplatin-induced apoptosis. *Int J Oncol* **22:** (3) 663.
Ishida S, Shigemoto-Mogami Y, Kagechika H, Shudo K, Ozawa S, Sawada J, Ohno Y, Inoue K. 2003. Clinically potential subclasses of retinoid synergists revealed by gene expression profiling. *Mol Cancer Ther* **2:** (1) 49.
Jelinsky SA, Harris HA, Brown EL, Flanagan K, Zhang XC, Tunkey C, Lai KD, Lane MV, Simcoe DK, Evans MJ. 2003. Global transcription profiling of estrogen activity: Estrogen receptor a regulates gene expression in the kidney. *Endocrinology* **144:** (2) 701.
Katsuma S, Hada Y, Shiojima S, Hirasawa A, Tanoue A, Takagaki K, Ohgi T, Yano J, Tsujimoto G. 2003. Transcriptional profiling of gene expression patterns during sphingosine 1-phosphate-induced mesangial cell proliferation. *Biochem Biophys Res Commun* **300:** (2) 577.
Kitteringham NR, Powell H, Jenkins RE, Hamlett J, Lovatt C, Elsby R, Henderson CJ, Wolf CR, Pennington SR, Park BK. 2003. Protein expression profiling of glutathione S-transferase π null mice as a strategy to identify potential markers of resistance to paracetamol-induced toxicity in the liver. *Proteomics* **3:** (2) 191.
Liotta LA, Petricoin EF, Ardekani AM, Hitt BA, Levine PJ, Fusaro VA, Steinberg SM, Mills GB, Simone C, Fishman DA *et al*. 2003. Proteomic patterns in sera serve as biomarkers of ovarian cancer. *Gynecol Oncol* **88:** (1 Pt 2) S25.
Liu ET. 2003. Expression profiling and the study of ovarian cancer. *Gynecol Oncol* **88:** (1 Pt 2) S14.
Meireles SI, Carvalho AF, Hirata R, Montagnini AL, Martins WK, Runza FB, Stolf BS, Termini L, Neto CEM, Silva RLA *et al*. 2003. Differentially expressed genes in gastric tumors identified by cDNA array. *Cancer Lett* **190:** (2) 199.
Miller JC, Zhou HP, Kwekel J, Cavallo R, Burke J, Butler EB, Teh BS, Haab BB. 2003. Antibody microarray profiling of human prostate cancer sera: Antibody screening and identification of potential biomarkers. *Proteomics* **3:** (1) 56.
Moriyama M, Hoshida Y, Otsuka M, Nishimura S, Kato N, Goto T, Taniguchi H, Shiratori Y, Seki N, Omata M. 2003. Relevance network between chemosensitivity and transcriptome in human hepatoma cells. *Molec Canc Ther* **2:** (2) 199.
Mouledous L, Hunt S, Harcourt R, Harry JL, Williams KL, Gutstein HB. 2003. Proteomic analysis of immunostained, laser-capture microdissected brain samples. *Electrophoresis* **24:** (1-2) 296.
Onody A, Zvara A, Hackler L, Vigh L, Ferdinandy M, Puskas LG. 2003. Effect of classic preconditioning on the gene expression pattern of rat hearts: A DNA microarray study. *FEBS Lett* **536:** (1-3) 35.

Copyright © 2003 John Wiley & Sons, Ltd. 

Paulson L, Martin P, Persson A, Nilsson CL, Ljung E, Westman-Brinkmalm A, Eriksson PS, Blennow K, Davidsson P. 2003. Comparative genome- and proteome analysis of cerebral cortex from MK-801 treated rats. *J Neurosci Res* **71:** (4) 526.

Ramstrom M, Palmblad M, Markides KE, Hakansson P, Bergquist J. 2003. Protein identification in cerebrospinal fluid using packed capillary liquid chromatography Fourier transform ion cyclotron resonance mass spectrometry. *Proteomics* **3:** (2) 184.

Ringner M, Peterson C. 2003. Microarray-based cancer diagnosis with artificial neural networks. *Biotechniques* (Suppl) 30.

Scharpf R, Garrett ES, Hu J, Parmigiani G. 2003. Statistical modeling and visualization of molecular profiles in cancer. *Biotechniques* (Suppl) 22.

Seykora JT, Jih D, Elenitsas R, Horng WH, Elder DE. 2003. Gene expression profiling of melanocytic lesions. *Am J Dermatopathol* **25:** (1) 6.

Shin BK, Wang H, Yim AM, Le Naour F, Brichory F, Jang JH, Zhao R, Puravs E, Tra J, Michael CW *et al*. 2003. Global profiling of the cell surface proteome of cancer cells uncovers an abundance of proteins with chaperone function. *J Biol Chem* **278:** (9) 7607.

Szkanderova S, Hernychova L, Kasalova I, Vavrova J, Stulik J, Abend M, Van Beuningen D. 2003. Proteomic analysis of radiation-induced alterations in L929 cells. *Folia Biol (Krakow)* **49:** (1) 15.

Tellgren A, Wood TJJ, Flores-Morales A, Torndal UB, Eriksson L, Norstedt G. 2003. Differentially expressed transcripts in neoplastic hepatic nodules and neonatal rat liver studied by cDNA microarray analysis. *Int J Cancer* **104:** (2) 131.

Unwin RD, Harnden P, Pappin D, Rahman D, Whelan P, Craven RA, Selby PJ, Banks RE. 2003. Serological and proteomic evaluation of antibody responses in the identification of tumor antigens in renal cell carcinoma. *Proteomics* **3:** (1) 45.

Van Dyk DD, Misztal DR, Wilkins MR, Mackintosh JA, Poljak A, Varnail JC, Teber E, Walsh BJ, Gray PP. 2003. Identification of cellular changes associated with increased production of human growth hormone in a recombinant Chinese hamster ovary cell line. *Proteomics* **3:** (2) 147.

Waring JF, Gum R, Morfitt D, Jolly RA, Ciurlionis R, Heindel M, Gallenberg L, Buratto B, Ulrich RG. 2002. Identifying toxic mechanisms using DNA microarrays: Evidence that an experimental inhibitor of cell adhesion molecule expression signals through the aryl hydrocarbon nuclear receptor. *Toxicology* **181-182:** 537.

Watters JW, McLeod HL. 2003. Using genome-wide mapping in the mouse to identify genes that influence drug response. *Trends Pharmacol Sci* **24:** (2) 55.

Weekes J, Morrison K, Mullen A, Wait R, Barton P, Dunn MJ. 2003. Hyperubiquitination of proteins in dilated cardiomyopathy. *Proteomics* **3:** (2) 208.

Welss T, Papoutsaki M, Michel G, Reifenberger J, Chimenti S, Ruzicka T, Abts HF. 2003. Molecular basis of basal cell carcinoma: Analysis of differential gene expression by differential display PCR and expression array. *Int J Cancer* **104:** (1) 66.

Yun J, Zuscik MJ, Gonzalez-Cabrera P, McCune DF, Ross SA, Galvin R, Piascik MT, Perez DM. 2003. Gene expression profiling of $\alpha_{1b}$-adrenergic receptor-induced cardiac hypertrophy by oligonucleotide arrays. *Cardiovasc Res* **57:** (2) 443.

Zhan FH, Tian EM, Bumm K, Smith R, Barlogie B, Shaughnessy J. 2003. Gene expression profiling of human plasma cell differentiation and classification of multiple myeloma based on similarities to distinct stages of late-stage B-cell development. *Blood* **101:** (3) 1128.

Zhou J, Zhao LQ, Xiong MM, Wang XQ, Yang GR, Qui ZL, Wu M, Liu ZH. 2003. Gene expression profiles at different stages of human esophageal squamous cell carcinoma. *World J Gastroenterol* **9:** (1) 9.

### 8 EST, cDNA and other clone resources

Bellin D, Werber M, Theis T, Schulz B, Weisshaar B, Schneider K. 2002. EST sequencing, annotation and macroarray transcriptome analysis identify preferentially root-expressed genes in sugar beet. *Plant Biol* **4:** (6) 700.

Chang YL, Henriquez X, Preuss D, Copenhaver GP, Zhang HB. 2003. A plant-transformation-competent BIBAC library from the *Arabidopsis thaliana* Landsberg ecotype for functional and comparative genomics. *Theor Appl Genet* **106:** (2) 269.

Fischer K, Holt DC, Wilson P, Davis J, Hewitt V, Johnson M, McGrath A, Currie BJ, Walton SF, Kemp DJ. 2003. Normalization of a cDNA library cloned in λZAP by a long PCR and cDNA reassociation procedure. *Biotechniques* **34:** (2) 250.

Ronning CM, Stegalkina SS, Ascenzi RA, Bougri O, Hart AL, Utterbach TR, Vanaken SE, Riedmuller SB, White JA, Cho J *et al*. 2003. Comparative analyses of potato expressed sequence tag libraries. *Plant Physiol* **131:** (2) 419.

Sahi C, Agarwal M, Reddy MK, Sopory SK, Grover A. 2003. Isolation and expression analysis of salt stress-associated ESTs from contrasting rice cultivars using a PCR-based subtraction method. *Theor Appl Genet* **106:** (4) 620.

Shrager J, Hauser C, Chang CW, Harris EH, Davies J, McDermott J, Tamse R, Zhang ZD, Grossman AR. 2003. *Chlamydomonas reinhardtii* genome project. A guide to the generation and use of the cDNA information. *Plant Physiol* **131:** (2) 401.

Wu-Scharf D, Scharf ME, Pittendrigh BR, Bennett GW. 2003. Expressed sequence tags from a polyphenic *Reticulitermes flavipes* (Isoptera: Rhinotermitidae) cDNA library. *Sociobiology* **41:** (2) 479.

### 9 Functional genomics

Abler LL, Sheets MD. 2003. Expression of scFv antibodies in *Xenopus* embryos to disrupt protein function: Implications for large-scale evaluation of the embryonic proteome. *Genesis* **35:** (2) 107.

Amagai M, Ariizumi T, Endo M, Hatakeyama K, Kuwata C, Shibata D, Toriyama K, Watanabe M. 2003. Identification of anther-specific genes in a cruciferous model plant, *Arabidopsis thaliana*, by using a combination of *Arabidopsis* macroarray and mRNA derived from *Brassica oleracea*. *Sex Plant Reprod* **15:** (5) 213.

Blackburn AS, Avery SV. 2003. Genome-wide screening of *Saccharomyces cerevisiae* to identify genes required for antibiotic insusceptibility of eukaryotes. *Antimicrob Agents Chemother* **47:** (2) 676.

Buschlen S, Amillet JM, Guiard B, Fournier A, Marcireau C, Bolotin-Fukuhara M. 2003. The *S. cerevisiae* HAP complex, a key regulator of mitochondrial function, coordinates nuclear and mitochondrial gene expression. *Comp Funct Genom* **4:** (1) 37.

Chen C, Grzegorzewski KJ, Barash S, Zhao QH, Schneider H, Singh M, Pukac L, Bell AC, Duan R, Coleman T *et al*. 2003. An integrated functional genomics screening program reveals a role for BMP-9 in glucose homeostasis. *Nat Biotechnol* **21:** (3) 294.

Enyenihi AH, Saunders WS. 2003. Large-scale functional genomic analysis of sporulation and meiosis in *Saccharomyces cerevisiae*. *Genetics* **163:** (1) 47.

Hirai MY, Fujiwara T, Awazuhara M, Kimura T, Noji M, Saito K. 2003. Global expression profiling of sulfur-starved *Arabidopsis* by DNA macroarray reveals the role of *O*-acetyl-L-serine as a general regulator of gene expression in response to sulfur nutrition. *Plant J* **33:** (4) 651.

Martinez IM, Chrispeels MJ. 2003. Genomic analysis of the unfolded protein response in *Arabidopsis* shows its connection to important cellular processes. *Plant Cell* **15:** (2) 561.

Millar AH, Heazlewood JL. 2003. Genomic and proteomic analysis of mitochondrial carrier proteins in *Arabidopsis*. *Plant Physiol* **131:** (2) 443.

Neiman PE, Grbic JJ, Polony TS, Kimmel R, Bowers SJ, Delrow J, Beeman KL. 2003. Functional genomic analysis reveals distinct neoplastic phenotypes associated with c-*myb* mutation in the bursa of Fabricius. *Oncogene* **2:** (7) 1073.

Ozawa T, Sako Y, Sato M, Kitamura T, Umezawa Y. 2003. A genetic approach to identifying mitochondrial proteins. *Nat Biotechnol* **21:** (3) 287.

Pothof J, Van Haaften G, Thijssen K, Kamath RS, Fraser AG, Ahringer J, Plasterk RHA, Tijsterman M. 2003. Identification of genes that protect the *C. elegans* genome against mutations by genome-wide RNAi. *Gene Dev* **17:** (4) 443.

Puthoff DP, Nettleton D, Rodermel SR, Baum TJ. 2003. *Arabidopsis* gene expression changes during cyst nematode parasitism revealed by statistical analyses of microarray expression profiles. *Plant J* **33:** (5) 911.

Rao PSS, Lim TM, Leung KY. 2003. Functional genomics approach to the identification of virulence genes involved in *Edwardsiella tarda* pathogenesis. *Infect Immun* **71:** (3) 1343.

Yoon SH, Han MJ, Lee SY, Jeong KJ, Yoo JS. 2003. Combined transcriptome and proteome analysis of *Escherichia coli* during high cell density culture. *Biotechnol Bioeng* **81:** (7) 753.

Copyright © 2003 John Wiley & Sons, Ltd. 

You MK, Hur CG, Ahn YS, Suh MC, Jeong BC, Shin JS, Bae JM. 2003. Identification of genes possibly related to storage root induction in sweetpotato. *FEBS Lett* **536:** (1-3) 101.

### 10 Transcriptomics

Arimoto T, Katagiri T, Oda K, Tsunoda T, Yasugi T, Osuga Y, Yoshikawa H, Nishii O, Yano T, Taketani Y *et al*. 2003. Genome-wide cDNA microarray analysis of gene-expression profiles involved in ovarian endometriosis. *Int J Oncol* **22:** (3) 551.

Berges H, Lauber E, Liebe C, Batut J, Kahn D, De Bruijn FJ, Ampe F. 2003. Development of *Sinorhizobium meliloti* pilot macroarrays for transcriptome analysis. *Appl Environ Microbiol* **69:** (2) 1214.

Bhalerao R, Keskitalo J, Sterky F, Erlandsson R, Bjorkbacka H, Birve SJ, Karlsson J, Gardestrom P, Gustafsson P, Lundeberg J *et al*. 2003. Gene expression in autumn leaves. *Plant Physiol* **131:** (2) 430.

Boer VM, De Winde JH, Pronk JT, Piper MDW. 2003. The genome-wide transcriptional responses of *Saccharomyces cerevisiae* growth on glucose in aerobic chemostat cultures limited for carbon, nitrogen, phosphorus, or sulfur. *J Biol Chem* **278:** (5) 3265.

Boheler KR, Volkova M, Morrell C, Garg R, Zhu Y, Marguiles K, Seymour AM, Lakatta EG. 2003. Sex-and age-dependent human transcriptome variability: Implications for chronic heart failure. *Proc Natl Acad Sci U S A* **100:** (5) 2754.

Chhabra SR, Shockley KR, Conners SB, Scott KL, Wolfinger RD, Kelly RM. 2003. Carbohydrate-induced differential gene expression patterns in the hyperthermophilic bacterium *Thermotoga maritima*. *J Biol Chem* **278:** (9) 7540.

Firoved AM, Deretic V. 2003. Microarray analysis of global gene expression in mucoid *Pseudomonas aeruginosa*. *J Bacteriol* **185:** (3) 1071.

Herrero J, Diaz-Uriarte R, Dopazo J. 2003. An approach to inferring transcriptional regulation among genes from large-scale expression data. *Comp Funct Genom* **4:** (1) 148.

Hihara Y, Sonoike K, Kanehisa M, Ikeuchi M. 2003. DNA microarray analysis of redox-responsive genes in the genome of the cyanobacterium *Synechocystis* sp strain PCC 6803. *J Bacteriol* **185:** (5) 1719.

James TC, Campbell S, Donnelly D, Bond U. 2003. Transcription profile of brewery yeast under fermentation conditions. *J Appl Microbiol* **94:** (3) 432.

Kimura M, Yamamoto YY, Seki M, Sakurai T, Sato M, Abe T, Yoshida S, Manabe K, Shinozaki K, Matsui M. 2003. Identification of *Arabidopsis* genes regulated by high light-stress using cDNA microarray. *Photochem Photobiol* **77:** (2) 226.

Kocabas AM, Li P, Cao DF, Karsi A, He CB, Patterson A, Ju ZL, Dunham RA, Liu ZJ. 2002. Expression profile of the channel catfish spleen: Analysis of genes involved in immune functions. *Mar Biotechnol* **4:** (6) 526.

Kurz S, Hubner C, Aepinus C, Theiss S, Guckenberger M, Panzner U, Weber J, Frosch M, Dietrich G. 2003. Transcriptome-based antigen identification for *Neisseria meningitidis*. *Vaccine* **21:** (7-8) 768.

Lin JH, Ozeki M, Javel E, Zhao ZF, Pan W, Schlentz E, Levine S. 2003. Identification of gene expression profiles in rat ears with cDNA microarrays. *Hear Res* **175:** (1-2) 2.

Lopez IP, Marti A, Milagro FI, Zulet MDA, Moreno-Aliaga MJ, Martinez JA, De Miguel C. 2003. DNA microarray analysis of genes differentially expressed in diet-induced (cafeteria) obese rats. *Obes Res* **11:** (2) 188.

Maguire TL, Grimmond S, Forrest A, Iturbe-Ormaetxe I, Meksem K, Gresshoff P. 2002. Tissue-specific gene expression in soybean (*Glycine max*) detected by cDNA microarray analysis. *J Plant Physiol* **159:** (12) 1361.

Musarrat J, Hashsham SA. 2003. Customized cDNA microarray for expression profiling of environmentally important genes of *Pseudomonas stutzeri* strain KC. *Teratog Carcinog Mutagen* (Suppl 1) 283.

Pillai B, Verma J, Abraham A, Francis P, Kumar Y, Tatu U, Brahmachari SK, Sadhale PP. 2003. Whole genome expression profiles of yeast RNA polymerase II core subunit, Rpb4, in stress and nonstress conditions. *J Biol Chem* **278:** (5) 3339.

Winzeler EA, Castillo-D'Avis CI, Oshiro G, Liang D, Richards DR, Zhou YY, Hartl DL. 2003. Genetic diversity in yeast assessed with whole-genome oligonucleotide arrays. *Genetics* **163:** (1) 79.

Xu Q, Dziejman M, Mekalanos JJ. 2003. Determination of the transcriptome of *Vibrio cholerae* during intraintestinal growth and midexponential phase *in vitro*. *Proc Natl Acad Sci U S A* **100:** (3) 1286.

### 11 Proteomics

Allen S, Heath PR, Kirby J, Wharton SB, Cookson MR, Menzies FM, Banks RE, Shaw PJ. 2003. Analysis of the cytosolic proteome in a cell culture model of familial amyotrophic lateral sclerosis reveals alterations to the proteasome, antioxidant defenses, and nitric oxide synthetic pathways. *J Biol Chem* **278:** (8) 6371.

Basrur V, Yang F, Kushimoto T, Higashimoto Y, Yasumoto K, Valencia J, Muller J, Vieira WD, Watabe H, Shabanowitz J *et al*. 2003. Proteomic analysis of early melanosomes: Identification of novel melanosomal proteins. *J Proteome Res* **2:** (1) 69.

Bernhardt J, Weibezahn J, Scharf C, Hecker M. 2003. *Bacillus subtilis* during feast and famine: Visualization of the overall regulation of protein synthesis during glucose starvation by proteome analysis. *Genome Res* **13:** (2) 224.

Blagoev B, Kratchmarova I, Ong SE, Nielsen M, Foster LJ, Mann M. 2003. A proteomics strategy to elucidate functional protein-protein interactions applied to EGF signaling. *Nat Biotechnol* **21:** (3) 315.

Branlard G, Amiour N, Igrejas G, Gaborit T, Herbette S, Dardevet M, Marion D. 2003. Diversity of puroindolines as revealed by two-dimensional electrophoresis. *Proteomics* **3:** (2) 168.

Careri M, Elviri L, Mangia A, Zagnoni I, Agrimonti C, Visioli G, Marmiroli N. 2003. Analysis of protein profiles of genetically modified potato tubers by matrix-assisted laser desorption/ionization time-of-flight mass spectrometry. *Rapid Commun Mass Spectrom* **17:** (5) 479.

Cavusoglu N, Brand M, Tora L, Van Dorsselaer A. 2003. Novel subunits of the TATA binding protein free TAF(II)-containing transcription complex identified by matrix-assisted laser desorption/ionization-time of flight mass spectrometry following one-dimensional gel electrophoresis. *Proteomics* **3:** (2) 217.

Champion MM, Campbell CS, Siegele DA, Russell DH, Hu JC. 2003. Proteome analysis of *Escherichia coli* K12 by two-dimensional native-state chromatography and MALDI-MS. *Mol Microbiol* **47:** (2) 383.

Durrschmid K, Marzban G, Durrschmid E, Striedner G, Clementschitsch F, Cserjan-Puschmann L, Bayer K. 2003. Monitoring of protein profiles for the optimization of recombinant fermentation processes using public domain databases. *Electrophoresis* **24:** (1-2) 303.

Garin J. 2002. Proteomic analysis, cell exploration and annotation (French). *Biofutur* (Suppl 1) 28.

Gelfi C, De Palma S, Cerretelli P, Begum S, Wait R. 2003. Two-dimensional protein map of human vastus lateralis muscle. *Electrophoresis* **24:** (1-2) 286.

Herald VL, Heazlewood JL, Day DA, Millar AH. 2003. Proteomic identification of divalent metal cation binding proteins in plant mitochondria. *FEBS Lett* **537:** (1-3) 96.

Lai EM, Phadke ND, Kachman MT, Giorno R, Vazquez S, Vazquez JA, Maddock JR, Driks A. 2003. Proteomic analysis of the spore coats of *Bacillus subtilis* and *Bacillus anthracis*. *J Bacteriol* **185:** (4) 1443.

Liska AJ, Shevchenko A. 2003. Expanding the organismal scope of proteomics: Cross-species protein identification by mass spectrometry and its implications. *Proteomics* **3:** (1) 19.

Majoul T, Bancel E, Triboi E, Ben Hamida J, Branlard G. 2003. Proteomic analysis of the effect of heat stress on hexaploid wheat grain: Characterization of heat-responsive proteins from total endosperm. *Proteomics* **3:** (2) 175.

Mawuenyega KG, Kaji H, Yamauchi Y, Shinkawa T, Saito H, Taoka M, Takahashi N, Isobe T. 2003. Large-scale identification of *Caenorhabditis elegans* proteins by multidimensional liquid chromatography-tandem mass spectrometry. *J Proteome Res* **2:** (1) 23.

Repetto O, Bestel-Corre G, Dumas-Gaudot E, Berta G, Gianinazzi-Pearson V, Gianinazzi S. 2003. Targeted proteomics to identify cadmium-induced protein modifications in *Glomus mosseae*-inoculated pea roots. *New Phytol* **157:** (3) 555.

Romer TG, Boyle MDP. 2003. Application of immunoproteomics to analysis of post-translational processing of the antiphagocytic M protein of *Streptococcus*. *Proteomics* **3:** (1) 29.

Salujarvi L, Poutanen M, Pitkanen JP, Koivistoinen H, Aristidou A, Kalkkinen N, Ruohonen L, Penttila M. 2003. Proteome analysis of recombinant xylose-fermenting *Saccharomyces cerevisiae*. *Yeast* **20:** (4)

Copyright © 2003 John Wiley & Sons, Ltd.

295.
Shen SH, Sharma A, Komatsu S. 2003. Characterization of proteins responsive to gibberellin in the leaf-sheath of rice (*Oryza sativa* L) seedling using proteome analysis. *Biol Pharm Bull* **26:** (2) 129.
Smolka MB, Martins D, Winck FV, Santoro CE, Castellari RR, Ferrari F, Brum IJ, Galembeck E, Coletta HD, Machado MA *et al*. 2003. Proteome analysis of the plant pathogen *Xylella fastidiosa* reveals major cellular and extracellular proteins and a peculiar codon bias distribution. *Proteomics* **3:** (2) 224.
Tahara ST, Mehta A, Rosato YB. 2003. Proteins induced by *Xanthomonas axonopodis* pv. *passiflorae* with leaf extract of the host plant (*Passiflorae edulis*). *Proteomics* **3:** (1) 95.
Taylor CF, Paton NW, Garwood KL, Kirby PD, Stead DA, Yin ZK, Deutsch EW, Selway L, Walker J, Riba-Garcia I *et al*. 2003. A systematic approach to modeling, capturing, and disseminating proteomics experimental data. *Nat Biotechnol* **21:** (3) 247.
Taylor SW, Fahy E, Zhang B, Glenn GM, Warnock DE, Wiley S, Murphy AN, Gaucher SP, Capaldi RA, Gibson BW *et al*. 2003. Characterization of the human heart mitochondrial proteome. *Nat Biotechnol* **21:** (3) 281.
Trabalzini L, Paffetti A, Scaloni A, Talamo F, Ferro E, Coratza G, Bovalini L, Lusini P, Martelli P, Santucci A. 2003. Proteomic response to physiological fermentation stresses in a wild-type wine strain of *Saccharomyces cerevisiae*. *Biochem J* **370:** (1) 35.
Yao Y, Berg EA, Costello CE, Troxler RF, Oppenheim FG. 2003. Identification of protein components in human acquired enamel pellicle and whole saliva using novel proteomics approaches. *J Biol Chem* **278:** (7) 5300.
Ying WT, Zhang KT, Qian XH, Xie L, Wang J, Xiang XQ, Cai Y, Wu DC. 2003. Proteome analysis on an early transformed human bronchial epithelial cell line, BEP2D, after α-particle irradiation. *Proteomics* **3:** (1) 64.
Zischka H, Keller H, Kellermann J, Eckerskorn C, Schuster SC. 2003. Proteome analysis of the thermoreceptive pit membrane of the western diamondback rattlesnake *Crotalus atrox*. *Proteomics* **3:** (1) 78.
Zolla L, Timperio AM, Walcher W, Huber CG. 2003. Proteomics of light-harvesting proteins in different plant species. Analysis and comparison by liquid chromatography-electrospray ionization mass spectrometry. Photosystem II. *Plant Physiol* **131:** (1) 198.

### 12 Protein structural genomics

Quevillon-Cheruel S, Collinet B, Zhou CZ, Minard P, Blondeau K, Henkes G, Aufrere R, Coutant J, Guittet E, Lewit-Bentley A *et al*. 2003. A structural genomics initiative on yeast proteins. *J Synchrotron Radiat* **10:** (1) 4.
Sali A, Glaeser R, Earnest T, Baumeister W. 2003. From words to literature in structural proteomics. *Nature* **422:** (6928) 216.
Savchenko A, Yee A, Khachatryan A, Skarina T, Evdokimova E, Pavlova M, Semesi A, Northey J, Beasley S, Lan N *et al*. 2003. Strategies for structural proteomics of prokaryotes: Quantifying the advantages of studying orthologous proteins and of using both NMR and X-ray crystallography approaches. *Protein-Struct Funct Genet* **50:** (3) 392.
Tcherkasskaya O, Davidson EA, Uversky VN. 2003. Biophysical constraints for protein structure prediction. *J Proteome Res* **2:** (1) 37.
Yang JK, Park MS, Waldo GS, Suh SW. 2003. Directed evolution approach to a structural genomics project: Rv2002 from *Mycobacterium tuberculosis*. *Proc Natl Acad Sci U S A* **100:** (2) 455.

### 13 Metabolomics

Dilks K, Rose RW, Hartmann E, Pohlschroder M. 2003. Prokaryotic utilization of the twin-arginine translocation pathway: A genomic survey. *J Bacteriol* **185:** (4) 1478.
Forster J, F'Amili I, Fu P, Palsson BO, Nielsen J. 2003. Genome-scale reconstruction of the *Saccharomyces cerevisiae* metabolic network. *Genome Res* **13:** (2) 244.
Maharjan RP, Ferenci T. 2003. Global metabolite analysis: The influence of extraction methodology on metabolome profiles of *Escherichia coli*. *Anal Biochem* **313:** (1) 145.
Nikiforova V, Freitag J, Kempa S, Adamik M, Hesse H, Hoefgen R. 2003. Transcriptome analysis of sulfur depletion in *Arabidopsis thaliana*: Interlacing of biosynthetic pathways provides response specificity. *Plant J* **33:** (4) 633.
Zazopoulos E, Huang KX, Staffa A, Liu W, Bachmann BO, Nonaka K, Ahlert J, Thorson JS, Shen B, Farnet CM. 2003. A genomics-guided approach for discovering and expressing cryptic metabolic pathways. *Nat Biotechnol* **21:** (2) 187.
Zhang W, Needham DL, Coffin M, Rooker A, Hurban P, Tanzer MM, Shuster JR. 2003. Microarray analyses of the metabolic responses of *Saccharomyces cerevisiae* to organic solvent dimethyl sulfoxide. *J Ind Microbiol Biotechnol* **30:** (1) 57.

### 14 Genomic approaches to development

Butler MJ, Jacobsen TL, Cain DM, Jarman MG, Hubank M, Whittle JRS, Phillips R, Simcox A. 2003. Discovery of genes with highly restricted expression patterns in the *Drosophila* wing disc using DNA oligonucleotide microarrays. *Development* **130:** (4) 659.
Fowles LF, Bennetts JS, Berkman JL, Williams E, Koopman P, Teasdale RD, Wicking C. 2003. Genomic screen for genes involved in mammalian craniofacial development. *Genesis* **35:** (2) 73.
Ishino T, Shirai M, Kunieda T, Sekimizu K, Natori S, Kubo T. 2003. Identification of genes induced in regenerating *Xenopus* tadpole tails by using the differential display method. *Dev Dyn* **226:** (2) 317.
Jochheim A, Cieslak A, Hillemann T, Cantz T, Scharf J, Manns MP, Ott M. 2003. Multi-stage analysis of differential gene expression in BALB/C mouse liver development by high-density microarrays. *Differentiation* **71:** (1) 62.
Lee CY, Clough EA, Yellon P, Teslovich TM, Stephan DA, Baehrecke EH. 2003. Genome-wide analyses of steroid- and radiation- triggered programmed cell death in *Drosophila*. *Curr Biol* **13:** (4) 350.

### 15 Technological advances

Aebersold R, Mann M. 2003. Mass spectrometry-based proteomics. *Nature* **422:** (6928) 198.
Alban A, David SO, Bjorkesten L, Andersson C, Sloge E, Lewis S, Currie I. 2003. A novel experimental design for comparative two-dimensional gel analysis: Two-dimensional difference gel electrophoresis incorporating a pooled internal standard. *Proteomics* **3:** (1) 36.
Barrett JC, Kawasaki ES. 2003. Microarrays: The use of oligonucleotides and cDNA for the analysis of gene expression. *Drug Discov Today* **8:** (3) 134.
Beardsley RL, Reilly JP. 2003. Quantitation using enhanced signal tags: A technique for comparative proteomics. *J Proteome Res* **2:** (1) 15.
Bernhard OK, Burgess JA, Hochgrebe T, Sheil MM, Cunningham AL. 2003. Mass spectrometry analysis of CD4-associating proteins using affinity chromatography and affinity tag-mediated purification of tryptic peptides. *Proteomics* **3:** (2) 139.
Bhattacharya SH, Gal AA, Murray KK. 2003. Laser capture microdissection MALDI for direct analysis of archival tissue. *J Proteome Res* **2:** (1) 95.
Chen ZJJ, Gate L, Davis W, Ile KE, Tew KD. 2002. Integration of amplified differential gene expression (ADGE) and DNA microarray. *IUBMB Life* **54:** (6) 335.
Choudhary G, Wu SL, Shieh P, Hancock WS. 2003. Multiple enzymatic digestion for enhanced sequence coverage of proteins in complex proteomic mixtures using capillary LC with ion trap MS/MS. *J Proteome Res* **2:** (1) 59.
Cooper JW, Chen JZ, Li Y, Lee CS. 2003. Membrane-based nanoscale proteolytic reactor enabling protein digestion, peptide separation, and protein identification using mass spectrometry. *Anal Chem* **75:** (5) 1067.
Cordingley HC, Robets SLL, Tooke P, Armitage JR, Lane PW, Wu W, Wildsmith SE. 2003. Multifactorial screening design and analysis of SELDI-TOF ProteinChip® array optimization experiments. *Biotechniques* **34:** (2) 364.
Delehanty JB, Ligler FS. 2003. Method for printing functional protein microarrays. *Biotechniques* **34:** (2) 380.
Dennis P, Edwards EA, Liss SN, Fulthorpe R. 2003. Monitoring gene expression in mixed microbial communities by using DNA microarrays. *Appl Environ Microbiol* **69:** (2) 769.
Draghici S, Khatri P, Shah A, Tainsky MA. 2003. Assessing the functional bias of commercial microarrays using the onto-compare database. *Biotechniques* (Suppl) 55.

Copyright © 2003 John Wiley & Sons, Ltd. 

Drobyshev AL, Machka C, Horsch M, Seltmann M, Liebscher V, De Angelis MH, Beckers J. 2003. Specificity assessment from fractionation experiments (SAFE): A novel method to evaluate microarray probe specificity based on hybridisation stringencies - Online article no. e1. *Nucleic Acids Res* **31:** (2) e1.

Giavalisco P, Nordhoff E, Lehrach H, Gobom J, Klose J. 2003. Extraction of proteins from plant tissues for two-dimensional electrophoresis analysis. *Electrophoresis* **24:** (1-2) 207.

Giorgianni F, Desiderio DM, Beranova-Giorgianni S. 2003. Proteome analysis using isoelectric focusing in immobilized pH gradient gels followed by mass spectrometry. *Electrophoresis* **24:** (1-2) 253.

Gorski SM, Chittaranjan S, Pleasance ED, Freeman JD, Anderson CL, Varhol RJ, Coughlin SM, Zuyderduyn SD, Jones SJM, Marra MA. 2003. A SAGE approach to discovery of genes involved in autophagic cell death. *Curr Biol* **13:** (4) 358.

Griffin TJ, Lock CM, Li XJ, Patel A, Chervetsova L, Lee H, Wright ME, Ranish JA, Chen SS, Aebersold R. 2003. Abundance ratio-dependent proteomic analysis by mass spectrometry. *Anal Chem* **75:** (4) 867.

Gu S, Pan SQ, Bradbury EM, Chen XA. 2003. Precise peptide sequencing and protein quantification in the human proteome through *in vivo* lysine-specific mass tagging. *J Am Spec Mass Spectrom* **14:** (1) 1.

Gupta V, Cherkassky A, Chatis P, Joseph R, Johnson AL, Broadbent J, Erickson T, Di Meo J. 2003. Directly labeled mRNA produces highly precise and unbiased differential gene expression data: Online article no. e13. *Nucleic Acids Res* **31:** (4) e13.

Hessner MJ, Wang XJ, Hulse K, Meyer L, Wu Y, Nye S, Guo SW, Ghosh S. 2003. Three color cDNA microarrays: Quantitative assessment through the use of fluorescein-labeled probes: Online article no. e14. *Nucleic Acids Res* **31:** (4) e14.

Hoyt PR, Tack L, Jones BH, Van Dinther J, Staat S, Doktycz MJ. 2003. Automated high-throughput probe production for DNA microarray analysis. *Biotechniques* **34:** (2) 402.

Irizarry RA, Bolstad BM, Collin F, Cope LM, Hobbs B, Speed TP. 2003. Summaries of affymetrix GeneChip probe level data: Online article no. e15. *Nucleic Acids Res* **31:** (4) e15.

Joubert-Caron R, Caron M. 2002. Differential analysis of proteins (French). *Biofutur* (Suppl 1) 20.

Kiss C, Nishikawa J, Dieckmann A, Takada K, Klein G, Szekely L. 2003. Improved subtractive suppression hybridization combined with high density cDNA array screening identifies differentially expressed viral and cellular genes. *J Virol Methods* **107:** (2) 195.

Li Y, De Voe DL, Lee CS. 2003. Dynamic analyte introduction and focusing in plastic microfluidic devices for proteomic analysis. *Electrophoresis* **24:** (1-2) 193.

Lu BW, Chen T. 2003. A suboptimal algorithm for *de novo* peptide sequencing *via* tandem mass spectrometry. *J Comput Biol* **10:** (1) 1.

Luzzi V, Mahadevappa M, Raja R, Warrington JA, Watson MA. 2003. Accurate and reproducible gene expression profiles from laser capture microdissection, transcript amplification, and high density oligonucleotide microarray analysis. *J Mol Diagn* **5:** (1) 9.

Martinez MJ, Aragon AD, Rodriguez AL, Weber JM, Timlin JA, Sinclair MB, Haaland DM, Werner-Washburne M. 2003. Identification and removal of contaminating fluorescence from commercial and in-house printed DNA microarrays: Online article no. e18. *Nucleic Acids Res* **31:** (4) e18.

Nakajima K, Oda Y, Kinoshita M, Kakehi K. 2003. Capillary affinity electrophoresis for the screening of post-translational modification of proteins with carbohydrates. *J Proteome Res* **2:** (1) 81.

Nandakumar MP, Shen J, Raman B, Marten MR. 2003. Solubilization of trichloroacetic acid (TCA) precipitated microbial proteins *via* NaOH for two-dimensional electrophoresis. *J Proteome Res* **2:** (1) 89.

Peluso P, Wilson DS, Do D, Tran H, Venkatasubbaiah M, Quincy D, Heidecker B, Poindexter K, Tolani N, Phelan M *et al*. 2003. Optimizing antibody immobilization strategies for the construction of protein microarrays. *Anal Biochem* **312:** (2) 113.

Peng JM, Elias JE, Thoreen CC, Licklider LJ, Gygi SP. 2003. Evaluation of multidimensional chromatography coupled with tandem mass spectrometry (LC/LS-MS/MS) for large-scale protein analysis: The yeast proteome. *J Proteome Res* **2:** (1) 43.

Petritis K, Kangas LJ, Ferguson PL, Anderson GA, Pasa-Tolic L, Lipton MS, Auberry KJ, Strittmatter EF, Shen YF, Zhao R *et al*. 2003. Use of artificial neural networks for the accurate prediction of peptide liquid chromatography elution times in proteome analyses. *Anal Chem* **75:** (5) 1039.

Phizicky E, Bastiaens PIH, Zhu H, Snyder M, Fields S. 2003. Protein analysis on a proteomic scale. *Nature* **422:** (6928) 208.

Pietrogrande MC, Marchetti N, Dondi F, Righetti PG. 2003. Spot overlapping in two-dimensional polyacrylamide gel electrophoresis maps: Relevance to proteomics. *Electrophoresis* **24:** (1-2) 217.

Rabilloud T, Luche S, Chevallet M. 2002. Gel electrophoresis techniques in proteomic analysis (French). *Biofutur* (Suppl 1) 11.

Raghavachari N, Bao YP, Li GS, Xie XY, Muller UR. 2003. Reduction of autofluorescence on DNA microarrays and slide surfaces by treatment with sodium borohydride. *Anal Biochem* **312:** (2) 101.

Ranish JA, Yi EC, Leslie DM, Purvine SO, Goodlett DR, Eng J, Aebersold R. 2003. The study of macromolecular complexes by quantitative proteomics. *Nat Genet* **33:** (3) 349.

Rappsilber J, Ishihama Y, Mann M. 2003. Stop and go extraction tips for matrix-assisted laser desorption/ionization, nanoelectrospray, and LC/MS sample pretreatment in proteomics. *Anal Chem* **75:** (3) 663.

Rubenstein K. 2003. Commercial aspects of microarray technology. *Biotechniques* (Suppl) 52.

Saeed AI, Sharov V, White J, Li J, Liang W, Bhagabati N, Braisted J, Klapa M, Currier T, Thiagarajan M *et al*. 2003. TM4: A free, open-source system for microarray data management and analysis. *Biotechniques* **34:** (2) 374.

Scrivener E, Barry R, Platt A, Calvert R, Masih G, Hextall P, Soloviev M, Terrett J. 2003. Peptidomics: A new approach to affinity protein microarrays. *Proteomics* **3:** (2) 122.

Shi L, Khandurina J, Ronai Z, Li BY, Kwan WK, Wang X, Guttman A. 2003. Micropreparative capillary gel electrophoresis of DNA: Rapid expressed sequence tag library construction. *Electrophoresis* **24:** (1-2) 86.

Simon RM, Dobbin K. 2003. Experimental design of DNA microarray experiments. *Biotechniques* (Suppl) 16.

Smith L, Underhill P, Pritchard C, Tymowska-Lalanne Z, Abdul-Hussein S, Hilton H, Winchester L, Williams D, Freeman T, Webb S *et al*. 2003. Single primer amplification (SPA) of cDNA for microarray expression analysis: Online article no. e9. *Nucleic Acids Res* **31:** (3) e9.

Stich N, Van Steen G, Schalkhammer T. 2003. Design and peptide-based validation of phage display antibodies for proteomic biochips. *Comb Chem High Throughput Scr* **6:** (1) 67.

Strittmatter EF, Rodriguez N, Smith RD. 2003. High mass measurement accuracy determination for proteomics using multivariate regression fitting: Application to electrospray ionization time-of-flight mass spectrometry. *Anal Chem* **75:** (3) 460.

Tastet C, Charmont S, Chevallet M, Luche S, Rabilloud T. 2003. Structure-efficiency relationships of zwitterionic detergents as protein solubilizers in two-dimensional electrophoresis. *Proteomics* **3:** (2) 111.

Terletski V, Schwarz S, Carnwath J, Niemann H. 2003. Subtracted restriction fingerprinting: A tool for bacterial genome typing. *Biotechniques* **34:** (2) 304.

Vainrub A, Pettitt BM. 2003. Surface electrostatic effects in oligonucleotide microarrays: Control and optimization of binding thermodynamics. *Biopolymers* **68:** (2) 265.

Wang J, Hu L, Hamilton SR, Coombes KR, Zhang W. 2003. RNA amplification strategies for cDNA microarray experiments. *Biotechniques* **34:** (2) 394.

Wiese R. 2003. Analysis of several fluorescent detector molecules for protein microarray use. *Luminescence* **18:** (1) 25.

Xiang CC, Chen M, Kozhich OA, Phan QN, Inman JM, Chen Y, Brownstein MJ. 2003. Probe generation directly from small numbers of cells for DNA microarray studies. *Biotechniques* **34:** (2) 386.

Xin W, Ma J, Huang DW. 2003. Construction of linear functional expression elements with DNA and RNA hybrid primers: A flexible and fast method for proteomics. *Biotechnol Lett* **25:** (3) 273.

Zhu WH, Vigh G. 2003. A family of single-isomer, sulfated γ-cyclodextrin chiral resolving agents for capillary electrophoresis: Octa(6-*O*-sulfo)-γ-cyclodextrin. *Electrophoresis* **24:** (1-2) 130.

### 16 Bioinformatics

Agrawal H. 2002. Extreme self-organization in networks constructed from gene expression data: Article no. 268702. *Phys Rev Lett* **89:** (26) 268702.

Alexandrov N, Shindyalov I. 2003. PDP: Protein domain parser. *Bioinformatics* **19:** (3) 429.

Anderle P, Duval M, Draghici S, Kuklin A, Littlejohn TG, Medrano JE,

Copyright © 2003 John Wiley & Sons, Ltd. 

Vilanova D, Roberts MA. 2003. Gene expression databases and data mining. *Biotechniques* (Suppl) 36.
Arakawa K, Mori K, Ikeda K, Matsuzaki T, Kobayashi Y, Tomita M. 2003. G-language Genome Analysis Environment: A workbench for nucleotide sequence data mining. *Bioinformatics* **19:** (2) 305.
Barker G, Batley J, O'Sullivan H, Edwards KJ, Edwards D. 2003. Redundancy based detection of sequence polymorphisms in expressed sequence tag data using autoSNP. *Bioinformatics* **19:** (3) 421.
Bekaert M, Bidou L, Denise A, Duchateau-Nguyen G, Forest JP, Froidevaux C, Hatin I, Rousset JP, Termier M. 2003. Towards a computational model for -1 eukaryotic frameshifting sites. *Bioinformatics* **19:** (3) 327.
Bolstad BM, Irizarry RA, Astrand M, Speed TP. 2003. A comparison of normalization methods for high density oligonucleotide array data based on variance and bias. *Bioinformatics* **19:** (2) 185.
Bray N, Dubchak I, Pachter L. 2003. AVID: A global alignment program. *Genome Res* **13:** (1) 97.
Chuang TJ, Lin WC, Lee HC, Wang CW, Hsiao KL, Wang ZH, Shieh D, Lin SC, Chang LY. 2003. A complexity reduction algorithm for analysis and annotation of large genomic sequences. *Genome Res* **13:** (2) 313.
Chung MH, Kim CK, Nahm K. 2003. Fractional populations in multiple gene inheritance. *Bioinformatics* **19:** (2) 256.
Clarke ND, Granek JA. 2003. Rank order metrics for quantifying the association of sequence features with gene regulation. *Bioinformatics* **19:** (2) 212.
De Brevern AG, Hazout S. 2003. 'Hybrid protein model' for optimally defining 3D protein structure fragments. *Bioinformatics* **19:** (3) 345.
De Jong H, Geiselmann J, Hernandez C, Page M. 2003. Genetic Network Analyzer: qualitative simulation of genetic regulatory networks. *Bioinformatics* **19:** (3) 336.
Dozmorov I, Centola M. 2003. An associative analysis of gene expression array data. *Bioinformatics* **19:** (2) 204.
Dudoit S, Gendeman RC, Quackenbush J. 2003. Open source software for the analysis of microarray data. *Biotechniques* (Suppl) 45.
Fang Z, Polacco M, Chen S, Schroeder S, Hancock D, Sanchez H, Coe E. 2003. cMAP: The comparative genetic map viewer. *Bioinformatics* **19:** (3) 416.
Giles PF, Soanes DM, Talbot NJ. 2003. A relational database for the discovery of genes encoding amino acid biosynthetic enzymes in pathogenic fungi. *Comp Funct Genom* **4:** (1) 4.
Glasbey CA, Ghazal P. 2003. Combinatorial image analysis of DNA microarray features. *Bioinformatics* **19:** (2) 194.
Hahn U. 2003. Turning informal thesauri into formal ontologies: A feasibility study on biomedical knowledge re-use. *Comp Funct Genom* **4:** (1) 94.
Harhay GP, Keele JW. 2003. Positional candidate gene selection from livestock EST databases using Gene Ontology. *Bioinformatics* **19:** (2) 249.
Hsiao W, Wan I, Jones SJ, Brinkman FSL. 2003. IslandPath: Aiding detection of genomic islands in prokaryotes. *Bioinformatics* **19:** (3) 418.
Hsieh MH, Hsu WC, Chiu SK, Tzeng CM. 2003. An efficient algorithm for minimal primer set selection. *Bioinformatics* **19:** (2) 285.
Huang XQ, Chao KM. 2003. A generalized global alignment algorithm. *Bioinformatics* **19:** (2) 228.
Jaffe DB, Butler J, Gnerre S, Mauceli E, Lindblad-Toh K, Mesirov JP, Zody MC, Lander ES. 2003. Whole-genome sequence assembly for mammalian genomes: Arachne 2. *Genome Res* **13:** (1) 91.
Klamt S, Stelling J, Ginkel M, Gilles ED. 2003. FluxAnalyzer: exploring structure, pathways, and flux distributions in metabolic networks on interactive flux maps. *Bioinformatics* **19:** (2) 261.
Kokocinski F, Wrobel G, Hahn M, Lichter P. 2003. QuickLIMS: Facilitating the data management for DNA-microarray fabrication. *Bioinformatics* **19:** (2) 283.
Le SY, Chen JH, Konings D, Maizel JV. 2003. Discovering well-ordered folding patterns in nucleotide sequences. *Bioinformatics* **19:** (3) 354.
Lefebvre A, Lecroq T, Dauchel H, Alexandre J. 2003. FORRepeats: Detects repeats on entire chromosomes and between genomes. *Bioinformatics* **19:** (3) 319.
Leunissen JAM. 2003. Chimera: Construction of chimeric sequences for phylogenetic analysis. *Bioinformatics* **19:** (2) 303.
Linge JP, Habeck M, Rieping W, Nilges M. 2003. ARIA: Automated NOE assignment and NMR structure calculation. *Bioinformatics* **19:** (2) 315.
Lio P. 2003. Statistical bioinformatic methods in microbial genome analysis. *Bioessays* **25:** (3) 266.
Ma HW, Zeng AP. 2003. Reconstruction of metabolic networks from genome data and analysis of their global structure for various organisms. *Bioinformatics* **19:** (2) 270.
Majoros WH, Subramanian GM, Yandell MD. 2003. Identification of key concepts in biomedical literature using a modified Markov heuristic. *Bioinformatics* **19:** (3) 402.
McCarthy EM, McDonald JF. 2003. LTR STRUC: A novel search and identification program for LTR retrotransposons. *Bioinformatics* **19:** (3) 362.
Medjahed D, Smythers GW, Powell DA, Stephens RM, Lemkin PF, Munroe DJ. 2003. VIRTUAL2D: A web-accessible predictive database for proteomics analysis. *Proteomics* **3:** (2) 129.
Minch E, De Rinaldis M, Weiss S. 2003. pathSCOUT™: Exploration and analysis of biochemical pathways. *Bioinformatics* **19:** (3) 431.
Morgenstern B, Goel S, Sczyrba A, Dress A. 2003. AltAVisT: Comparing alternative multiple sequence alignments. *Bioinformatics* **19:** (3) 425.
Mullikin JC, Ning ZM. 2003. The phusion assembler. *Genome Res* **13:** (1) 81.
Naef F, Socci ND, Magnasco M. 2003. A study of accuracy and precision in oligonucleotide arrays: Extracting more signal at large concentrations. *Bioinformatics* **19:** (2) 178.
Parkinson J, Blaxter M. 2003. SimiTri: visualizing similarity relationships for groups of sequences. *Bioinformatics* **19:** (3) 390.
Pedersen JS, Hein J. 2003. Gene finding with a hidden Markov model of genome structure and evolution. *Bioinformatics* **19:** (2) 219.
Pei JM, Sadreyev R, Grishin NV. 2003. PCMA: Fast and accurate multiple sequence alignment based on profile consistency. *Bioinformatics* **19:** (3) 427.
Poluliakh N, Takagi T, Nakai K. 2003. MELINA: Motif extraction from promoter regions of potentially co-regulated genes. *Bioinformatics* **19:** (3) 423.
Raychaudhuri S, Altman RB. 2003. A literature-based method for assessing the functional coherence of a gene group. *Bioinformatics* **19:** (3) 396.
Reiner A, Yekutieli D, Benjamini Y. 2003. Identifying differentially expressed genes using false discovery rate controlling procedures. *Bioinformatics* **19:** (3) 368.
Satagopan JM, Panageas KS. 2003. A statistical perspective on gene expression data analysis. *Stat Med* **22:** (3) 481.
Sawa T, Ohno-Machado L. 2003. A neural network-based similarity index for clustering DNA microarray data. *Comput Biol Med* **33:** (1) 1.
Schwacke R, Schneider A, Van der Graaff E, Fischer K, Catoni E, Desimone M, Frommer WB, Flugge UI, Kunze R. 2003. ARAMEMNON, a novel database for *Arabidopsis* integral membrane proteins. *Plant Physiol* **131:** (1) 16.
Schwartz S, Kent WJ, Smit A, Zhang Z, Baertsch R, Hardison RC, Haussler D, Miller W. 2003. Human-mouse alignments with BLASTZ. *Genome Res* **13:** (1) 103.
Sivakumaran S, Hariharaputran S, Mishra J, Bhalla US. 2003. The Database of Quantitative Cellular Signaling: Management and analysis of chemical kinetic models of signaling networks. *Bioinformatics* **19:** (3) 408.
Tardieu F. 2003. Virtual plants: Modelling as a tool for the genomics of tolerance to water deficit. *Trends Plant Sci* **8:** (1) 9.
Toyoda T, Konagaya A. 2003. KnowledgeEditor: A new tool for interactive modeling and analyzing biological pathways based on microarray data. *Bioinformatics* **19:** (3) 433.
Ursing BM. 2003. WiGID: wireless genome information database. *Bioinformatics* **19:** (3) 439.
Wang LS, Xu Y. 2003. SEGID: Identifying interesting segments in (multiple) sequence alignments. *Bioinformatics* **19:** (2) 297.
Yeh I, Karp PD, Noy NF, Altman RB. 2003. Knowledge acquisition, consistency checking and concurrency control for Gene Ontology (GO). *Bioinformatics* **19:** (2) 241.
You LC, Hoonlor A, Yin J. 2003. Modeling biological systems using Dynetica: A simulator of dynamic networks. *Bioinformatics* **19:** (3) 435.
Zhou YH, Liu JD. 2003. AVA: visual analysis of gene expression microarray data. *Bioinformatics* **19:** (2) 293.
Zupan B, Demsar J, Bratko I, Juvan P, Halter JA, Kuspa A, Shaulsky G. 2003. GenePath: A system for automated construction of genetic networks from mutant data. *Bioinformatics* **19:** (3) 383.

Copyright © 2003 John Wiley & Sons, Ltd. 
